# Supplementary material for: The Evolution and Characterization of the RNA Interference Pathways in Lophotrochozoa
Source: Genome Biol Evol. 2024 May 7;16(5):evae098. doi: 10.1093/gbe/evae098 (PMC11114477; doi:10.1093/gbe/evae098)
Supplement: evae098_Supplementary_Data [file evae098_supplementary_data.zip › Supplementary_materials.zip!/selected_miRNAs/DaRe_selected_miRNas/DaRe_NC_007131.7_34959.pdf]

Provisional ID	:	NC_007131.7_34959
Score total	:	227
Score for star read(s)	:	3.9
Score for read counts	:	220.4
Score for mfe	:	1.7
Score for randfold	:	1.6
Score for cons. seed	:	-0.6
Total read count	:	444
Mature read count	:	430
Loop read count	:	0
Star read count	:	14

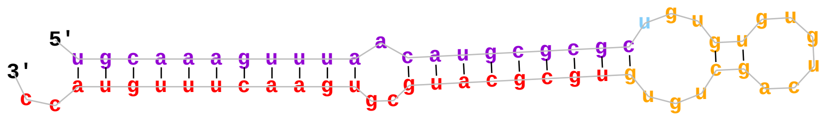

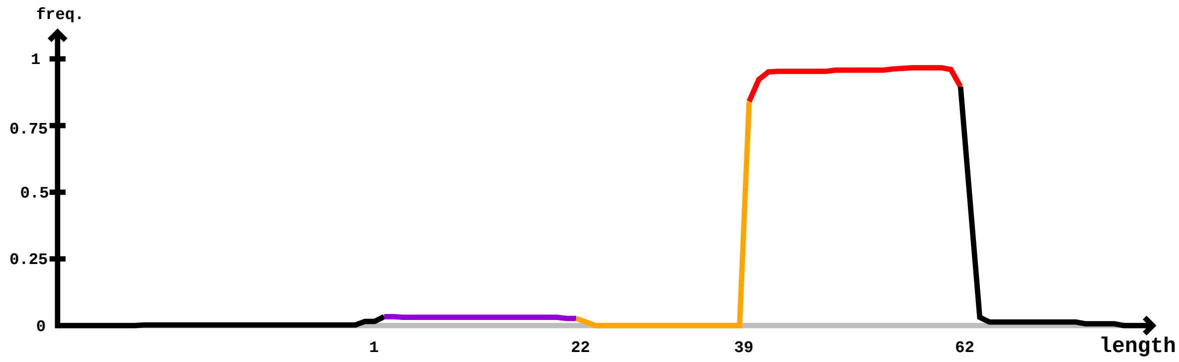

Star Mature

```
5'- uguauccugucacauuugccaugcaaaaacagugcaaaguuuaacaugcgcgcugugugugucagcugugugcgcaugcgugaacuuuguaccgacauugugugugacucau  -3'  obs
    uguauccugucacauuugccaugcaaaaacagugcaaaguuuaacaugcgcgcugugugugucagcugugugcgcaugcgugaacuuuguaccgacauugugugugacucau        exp
    ........(((((((.......((((...(.((((((((((((.(((((((((...((......))...)))))))))..)))))))))))).)...))))))))))).... reads   mm   sample
    ................................ugcaaaguuuaacaugcgcgc...........................................................  2    0    Gu2
    ......................................................................ugcgcaugcgugaacuuuguac....................  8    0    Gu2
    ......................................................................ugcgcaugcgugaacuuuguacU...................  10   1    Gu2
    ......................................................................ugcgcaugcgugaacuuuguacc...................  62   0    Gu2
    ......................................................................ugcgcaugcAugaacuuuguacc...................  1    1    Gu2
    ......................................................................ugcgcaugcgugaacuuuguacA...................  1    1    Gu2
    ......................................................................ugcgcaugcgugaacuuuguaccU..................  3    1    Gu2
    .......................................................................gcgcaugcgugaacuuuguac....................  1    0    Gu2
    .......................................................................gcgcaugcgugaacuuuguacc...................  7    0    Gu2
    .......................................................................gcgcaugcgugaacuuuguacU...................  5    1    Gu2
    ........................................................................cgcaugcgugaacuuuguaccga.................  3    0    Gu2
    ........................................................................cgcaugcgugaacuuuguaccgU.................  1    1    Gu2

    ..............................agugcaaaguuuaacaugcgcgc...........................................................  1    0    Ov3
    ................................ugcaaaguuuaacaugcgcgc...........................................................  2    0    Ov3
    ................................ugcaaaguuuaacaugcgcgcu..........................................................  1    0    Ov3
    ......................................................................ugcgcaugcgugaacuuuguacU...................  5    1    Ov3
    ......................................................................ugcgcaugcguAaacuuuguacc...................  1    1    Ov3
    ......................................................................ugcgcaugcgugaacuuuguacc...................  53   0    Ov3
    .......................................................................gcgcaugcgugaacuuuguacU...................  2    1    Ov3
    ........................................................................cgcaugcgugaacuuuguaccga.................  1    0    Ov3
    .........................................................................gcaugcgugaacuuuguaccga.................  1    0    Ov3
    .....................................................................................uuuguaAcgacauugugugugacu...  1    1    Ov3
    .......................................................................................uguaAcgacauugugugu.......  1    1    Ov3

    ..............................agugcaaaguuuaacaugcgc.............................................................  1    0    Gu1
    ..............................agAgcaaaguuuaacaugcgcgc...........................................................  1    1    Gu1
    ................................ugcaaaguuuaacaugcgcgcu..........................................................  1    0    Gu1
    ......................................................................ugcgcaugcgugaacuuugua.....................  2    0    Gu1
    ......................................................................ugcgcaugcgugaacuuuguac....................  9    0    Gu1
    ......................................................................ugcgcaugcgugaacuuuguacc...................  75   0    Gu1
    ......................................................................Ngcgcaugcgugaacuuuguacc...................  1    1    Gu1
    ......................................................................ugcgcaugcgugaacuuuUuacc...................  1    1    Gu1
```

```
                                Star                                    Mature
uguauccugucacauuugccaugcaaaaacagugcaaaguuuaacaugcgcgcugugugugucagcugugugcgcaugcgugaacuuuguaccgacauugugugugacucau
......................................................................Ggcgcaugcgugaacuuuguacc...................    1     1    Gu1
......................................................................ugcgcaugcgugaacuuuguacU...................    8     1    Gu1
......................................................................Cgcgcaugcgugaacuuuguacc...................    1     1    Gu1
......................................................................ugcgcaugcgugaacuuuguaccg..................    1     0    Gu1
......................................................................ugcgcaugcgugaacuuuguaccU..................    2     1    Gu1
.......................................................................gcgcaugcgugaacuuuguacc...................    1     0    Gu1
.......................................................................gcgcaugcgugaacuuuguacU...................    11    1    Gu1
.......................................................................gcgcaugcgugaacuuuguaccC..................    1     1    Gu1
.......................................................................gcgcaugcgugaacuuuguaccg..................    1     0    Gu1
.......................................................................gcgcaugcgugaacuuuguaccga.................    1     0    Gu1
........................................................................cgcaugcgugaacuuuguacc...................    3     0    Gu1
........................................................................cgcaugcgugaacuuuguaccg..................    1     0    Gu1

..............................agugcaaaguuuaacaugcgc.............................................................    1     0    Gu3
..............................agugcaaaguuuaacaugcgcgc...........................................................    1     0    Gu3
..............................agugcaaaguuuaacaugcgcgcu..........................................................    1     0    Gu3
................................ugcaaaguuuaacaugcgcgc...........................................................    1     0    Gu3
......................................................................ugcgcaugcgugaacuuugua.....................    1     0    Gu3
......................................................................ugcgcaugcgugaacuuuguac....................    7     0    Gu3
......................................................................ugcgcaugcgugaacuuuguacc...................    62    0    Gu3
......................................................................ugcgcaugcAugaacuuuguacc...................    1     1    Gu3
......................................................................ugcgcaugcgugaacuuuguacU...................    7     1    Gu3
......................................................................ugcgcGugcgugaacuuuguacc...................    1     1    Gu3
......................................................................ugcgcaugcgugaacuuuguaccU..................    4     1    Gu3
.......................................................................gcgcaugcgugaacuuuguacc...................    1     0    Gu3
.......................................................................gcgcaugcgugaacuuuguacU...................    1     1    Gu3
.......................................................................gcgcaugcgugaacuuuguaccg..................    1     0    Gu3
........................................................................cgcCugcgugaacuuuguacc...................    1     1    Gu3
........................................................................cgcaugcgAgaacuuuguacc...................    1     1    Gu3
........................................................................cgcaugcgugaacuuuguacc...................    1     0    Gu3
........................................................................cgcaugcgugaacuuuguaccga.................    1     0    Gu3

................................ugcaaaguuuaacaugcgcgcu..........................................................    1     0    Ov1
......................................................................ugcgcaugcgugaacuuuguac....................    1     0    Ov1
......................................................................ugcgcaugcgugaacuuuguacU...................    1     1    Ov1
......................................................................ugcgcaugcgugaacuuuguacc...................    18    0    Ov1
...............................................................................gugaacuuuguaAcgacauugugugu.......    2     1    Ov1
......................................................................................uuguaAcgacauugugugugacu...    1     1    Ov1

.......ugucacauuugccaugcaaaaacagug..............................................................................    1     0    Ov2
......................................................................ugcgcaugcgugaacuuuguac....................    3     0    Ov2
......................................................................ugcgcaugcgugaacuuuguacc...................    23    0    Ov2
......................................................................ugcgcaugcgugaacuuuguacU...................    4     1    Ov2
......................................................................ugcgcaugcgugaacuuuguaccU..................    1     1    Ov2
.......................................................................gcgcaugcgugaacuuuguacU...................    1     1    Ov2
.......................................................................gcgcaugcgugaacuuuguacc...................    3     0    Ov2
.....................................................................................uuuguaAcgacauugugugugacu...    1     1    Ov2
```
